# The Utilization of *Pseudomonas taetrolens* to Produce Lactobionic Acid

**DOI:** 10.1007/s12010-014-1024-x

**Published:** 2014-07-01

**Authors:** Kamila Goderska, Artur Szwengiel, Zbigniew Czarnecki

**Affiliations:** Faculty of Food Technology and Nutrition, Institute of Food Technology of Plant Origin, Poznan University of Life Sciences, Poznan, Poland

**Keywords:** Lactobionic acid, Lactose, Whey, *Pseudomonas taetrolens*

## Abstract

Lactobionic acid is a relatively new product derived from lactose oxidation, with high potential applications as a bioactive compound. Conducted experiments confirmed that both the time and temperature influenced the production of lactobionic acid during bioconversion of lactose using the *Pseudomonas taetrolens* bacteria. The study also investigated the effect of inoculum concentration on the production of lactobionic acid as a result of oxidation of whey-derived lactose. The highest concentration of lactobionic acid during oxidation of whey-derived lactose at a temperature of 30 °C by microorganisms. *P. taetrolens* was obtained during 50-h oxidation of the medium, which contained 25 % addition of the inoculum, in which the count of live cells was 2.85 × 10^9^ CFU/ml.

## Introduction

Lactobionic acid (LBA) is a relatively new product derived from lactose oxidation, with high potential applications as a bioactive compound.

Lactobionic acid is an aldonic acid obtained from the oxidation of lactose, with the high potential application as an ingredient in foods and pharmaceutical products because of its antioxidant, chelating and humectant properties [1]. The chemical structure of lactobionic acid comprise a galactose moiety linked to a gluconic acid molecule via an ether-like linkage.

Lactobionic acid is also used in calcium supplementation and represents a new ingredient in skin care products featuring potent antioxidant and humectant properties. In the food industry, lactobionic acid can be used as an acidulant with a sweet taste; as filler in cheese production; as firming agent, and to fortify functional drinks with essential minerals such as Fe and Cu [4].

Lactobionic acid is of value for medical and cosmetic purposes. Because of its efficient metal-chelating properties, lactobionic acid reduces oxidative damage to tissues during storage. Hence, it is used as a major component of organ preservation solutions during transplantation [4].

*Zymomonas mobilis*, a strictly fermentative gramme-negative ethanologenic bacterium, obtains its metabolic energy anaerobically via the Entner-Doudoroff pathway [2]. The ability of *Z. mobilis* to grow on high glucose concentration was originally explained by a rapid equilibration of the external and internal glucose concentrations achieved by the glucose facilitator system. The ability of *Z. mobilis* to counteract detrimental osmotic effects when grow on sucrose or mixtures of glucose and fructose has been attributed to the formation of sorbitol as a result of the activity of glucose–fructose oxidoreductase (GFOR).

Lactose is the only disaccharide present in milk and also in whey; it is a cheap industrial product obtained in abundant tonnage [3]. Recent papers by Druliolle et al., have shown that the electrocatalytic oxidation of lactose on noble metal electrodes in alkaline media permits us to form lactobionic acid with a high selectivity. Electrolysis carried out on Au electrodes showed that the conversation yield decreases when the initial concentration of lactose increase [3].

Lactose is mainly used as an ingredient in foods, beverages and confectionery products, and it has been extensively employed as diluent in tablets and carrier of medicines in the pharmaceutical industry. Nevertheless, the use of lactose is limited in many applications, because of its low sweetness and solubility, as well as due to the intolerance of some population segments, and only a small amount of lactose is employed as a raw material for producing fine chemicals [4].

However, the worldwide surplus and low cost of lactose have motivated research on innovative processes for producing valuable lactose derivatives, and expanding their applications in the food, pharmaceutical and chemical industries. Significant developments include the production of highly valued pharmaceutical products and functional food ingredients, such as lactitol, lactobionic acid (LBA), lactosucrose, lactulose and galacto-oligosaccharides, some of which have become commercially successful [5].

A new carbohydrate oxidase, lactose oxidase, with high specificity of oxidizing the disaccharide lactose to lactobionic acid has been found by Ahmad et al. 2004.

A programme of toxicological studies was conducted to establish the safety of lactose oxidase to be used as a processing aid in the food industry. The enzyme used in this study was produced by a submerged fermentation of *Fusarium venenatum* and contained a gene code from *Microdochium nivale* [6].

In food technology, lactobionic acid may find applications due to its ability to form mineral salt complexes and its presumed prebiotic effect. One of the new applications in focus is converting lactose in milk to lactobionic acid and exploiting the desirable characteristics of lactobionic acid to replace proteins and/or fats in process, cheeses and cream cheese. Lactobionic acid may even be seen as a flavour enhancer, a texture builder, and antioxidant synergist [6].

## Materials and Methods

### Microorganisms and Cultivation

#### Microorganism

*Pseudomonas taetrolens* DSM 21104 obtained from the Leibiniz-Institut DSMZ-German Collection of Microorganisms and Cell Cultures, was maintained frozen (in 40 % [*v*/*v*] glycerol at 20 °C). This strain was subsequently subcultured on Tryptone Soya (Casein soya bean digest) (Oxoid, England) agar plates, incubated for 48 h at 30 °C and then preserved at 4 °C.

#### Inoculum Preparation

A loopful of *P. taetrolens* from a fresh Tryptone Soya agar plate was used to inoculate a 500-mL Erlenmeyer flask containing 100-mL of Tryptone Soya broth medium. This flask was incubated on an orbital shaker at 250 rpm and 30 °C for 24 h. Active-growing cells from this culture were then employed as inoculum for the production of lactobionic acid in shake flasks and bioreactor seed cultures containing sweet whey, as subsequently reported.

#### Rennet Whey Preparation

Rennet whey (pH = 6.3, amount of lactose 30 mg/mL) (OSM Company TOP TOMYSL, Nowy Tomysl, Poland) was onefold diluted with distilled water (1:1) and adjusted to pH 6.5 (by adding NaOH 6 N) prior to sterilization using a tangential microfiltration device equipped with a PVDF membrane cassette of 0.22-μm pore size (Millipore, MA, USA).

### Preparative Scale Batch Reactions

Batch cultivations were performed in a 2-L bioreactor (Biostat®B, B. Braun Biotech International, Germany) with 1 L of whey as working volume, with aeration at 1 vol^−1^ min^−1^ and agitation at 120 rpm at 30 °C. Bioreactor experiments with an inoculation level of 10 % (*v*/*v*) were conducted at 30 °C. The bioreactor was equipped with a pH metre and a polarographic dissolved oxygen electrode in order to measure pH values online and continuously monitor dissolved oxygen tension (DOT), respectively. An efficient two-stage pH-shifted bioconversion strategy was adopted as previously described [7]: pH was controlled above 6.5 (pH was left uncontrolled above this value during the growth phase and subsequently maintained at 6.5) by means of computer-controlled peristaltic pumps via automatic addition of 2-M NaOH. These prior conditions were applied to all cultivations unless otherwise specified. Cultivations were carried out in duplicate as independent experiments.

### Disruption of Cells

Washed bacteria in acetic buffer (0.05 M, pH = 5.0) were disrupted by sonication (Polsonic, Palczynski Sp. J) at 80 W for a total time of 30 min. The temperature was kept below 4 ºC by placing the sonication vessel in an ice bucket filled with ice. Cell was removed by centrifugation at 15,000 *g* for 30 min at 4 °C. To obtain the high-speed supernatant fraction, cell-free extracts were centrifuged at 15,000 *g* for 30 min. This fraction was used either immediately or stored at −20 ºC.

### Estimation of Protein

The protein content of cell-free extracts and high-speed supernatants were determined by using the method of Bradford. Bovine serum albumin was employed as standard [8].

### Analysis of Lactose and Lactobionic Acid

Lactose and lactobionic acid were measured by Alliance HPLC (Waters) on Rezex ROA-Organic Acid column (300 × 7.8 mm; Phenomenex International, Torrance, CA, USA) at 210 nm - RI detector and PAD detector with an eluent of 0.025-M sulfuric acid, at a flow rate of 0.5 ml min^−1^. All samples were centrifuged to remove the cell mass and other water-insoluble substances, and then filtered through a 0.22-μm filter before the analysis.

### Characterization of the Production Microorganisms

The number of viable bacteria in each culture was determined by plate count on Tryptone Soya (Casein soya bean digest) (Oxoid, England) at 30 °C for 24 h. The number of live (CFU/ml) bacteria was determined using the Koch’s plate method [9].

## Results

Table 1 presents changes in the contents of lactose and lactobionic acid during oxidation of whey-derived lactose by enzymes produced by microorganisms *P. taetrolens*. The effect of inoculum concentration (5, 10, 15, 20 and 25 %) in the medium was analysed. Samples were collected immediately after inoculum was added to whey (0 h), as well as at 2, 4, 6, 9, 22, 24, 28, 32, 46 and 50 h, as presented in Table 1.

**Table 1:** Changes in contents of lactose and lactobionic acid during oxidation of whey-derived lactose by *Pseudomonas taetrolens* bacteria. The effect of inoculum concentration in the medium on the yield of lactobionic acid was analysed

Time (h)	Inoculum amount in medium [%]									
	5 %		10 %		15 %		20 %		25 %	
	Lactobionic acid [mg/ml]	Lactose [mg/ml]	Lactobionic acid [mg/ml]	Lactose [mg/ml]	Lactobionic acid [mg/ml]	Lactose [mg/ml]	Lactobionic acid [mg/ml]	Lactose [mg/ml]	Lactobionic acid [mg/ml]	Lactose [mg/ml]
0	1.04	30.29	1.16	28.20	1.77	30.06	1.51	23.58	3.43	29.68
2	0.63	34.00	0.83	34.94	1.10	29.04	0.77	32.21	1.45	15.34
4	1.16	31.53	1.38	29.67	0.92	28.27	1.60	23.57	1.90	26.60
6	1.46	28.85	2.27	37.22	1.77	25.22	2.01	26.14	2.02	21.32
9	2.54	27.75	2.63	27.76	2.55	23.76	2.84	23.15	3.22	23.33
22	6.45	23.98	6.89	23.31	3.81	15.91	4.24	19.66	4.95	21.06
24	6.91	23.86	7.71	24.78	5.57	21.25	5.44	20.65	6.05	22.32
28	8.20	23.80	9.64	25.25	5.28	18.34	5.49	18.93	7.63	21.80
32	9.51	25.18	10.70	25.13	7.89	23.85	7.68	23.11	9.03	19.29
46	12.99	24.19	17.01	23.14	13.47	21.98	13.23	19.44	17.94	18.60
50	15.79	24.05	19.31	23.28	14.82	20.88	15.85	19.34	20.85	17.16

The highest concentrations of lactobionic acid during oxidation of whey-derived lactose by microorganisms *P. taetrolens* were recorded at 50 h of oxidation in a medium, which contained 25 % added inoculum. The reaction was run at a temperature of 30 °C.

The experiment may be repeated, extending culture time in order to verify changes in contents of lactobionic acid and lactose, since in the presented results, the maximum content of lactobionic acid was recorded in the last hour of culture.

Moreover, the number of live microbial cells was determined using Koch’s plate method [9] at 24 h of culture. Inoculation results are presented below.

**Fig. 1:**
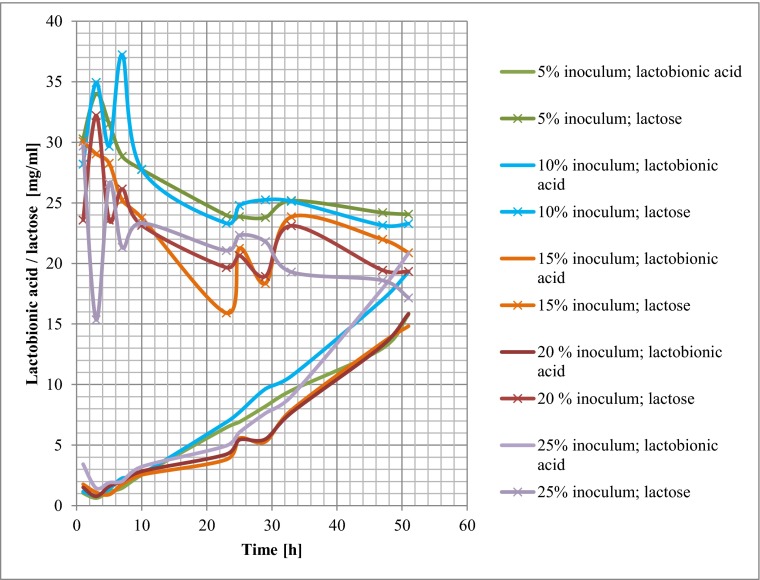
Changes in contents of lactose and lactobionic acid in culture with a 5, 10, 15, 20 and 25 % share of inoculum

Changes in contents of lactose and lactobionic acid during culture with different percentage concentrations of inoculum added to the medium are presented in Fig. 1. and Table 2.

**Table 2:** The number of live microbial cells determined at 24 h of culture with different concentrations of added inoculum [CFU/ml]

Inoculum amount (%)	Colony forming units [CFU/ml]
5	2.9 × 10^7^
10	3.2 × 10^8^
15	9.6 × 10^8^
20	1.39 × 10^9^
25	2.85 × 10^9^

The next experiment consisted in the stationary culture of microorganisms *P. taetrolens* in a bioreactor. Conditions found in the fermentation chamber were as follows: temperature 30 °C, pH 6.7, aeration rate 1 l/min and mixing at 120 rpm. The experiment was repeated three times. Results listed in Table 3 were averaged. Lactobionic acid was produced as a result of enzymatic oxidation of whey-derived lactose [7]. The highest content of lactobionic acid was obtained at 144 h of culture.

**Table 3:** Changes in contents of lactose and lactobionic acid during oxidation of whey-derived lactose by microorganisms *Pseudomonas taetrolens* (the number of culture I, II and III)

Time (h)	Lactobionic acid [mg/ml]				Lactose [mg/ml]			
	I	II	III	X	I	II	III	X
0	0.39	0.46	0.17	0.34	26.16	27.13	26.72	26.67
24	1.59	1.17	1.74	1.50	17.54	19.23	23.11	19.96
48	7.09	7.24	8.02	7.45	20.93	19.12	19.54	19.86
72	10.36	9.76	11.15	10.42	18.36	18.01	18.67	18.35
96	15.85	16.15	16.12	16.04	17.33	16.99	16.05	16.79
120	22.31	22.43	21.89	22.21	15.25	15.89	15.34	15.49
144	26.74	27.12	31.14	28.33	13.90	12.99	13.06	13.32

The number of live microbial cells was determined using Koch’s platelet method. Results of inoculations are presented in Tables 4, 5, 6 and 7

**Table 4:** Changes in *Pseudomonas taetrolens* counts during stationary culture run in a bioreactor

Time (h)	Counts of *Pseudomonas taetrolens* [CFU/ml]
0	5 × 10^7^
24	3.3 × 10^8^
48	3.4 × 10^8^
72	3.7 × 10^8^
96	3.8 × 10^8^
120	4.9 × 10^8^

**Table 5:** Changes in contents of lactose and lactobionic acid during oxidation of whey-derived lactose during continuous culture of microorganisms *Pseudomonas taetrolens* (the number of culture I, II, III).

Time (h)	Lactobionic acid [mg/ml]				Lactose [mg/ml]			
	I	II	III	X	I	II	III	X
0	0.55	0.58	0.53	0.55	35.23	33.57	37.30	35.37
12	1.89	2.01	1.82	1.91	33.76	32.17	35.74	33.89
24	5.73	6.09	5.54	5.79	30.32	28.89	32.10	30.44
36	8.37	8.90	8.09	8.45	27.70	26.40	29.33	27.81
48	8.58	9.11	8.28	8.66	27.27	25.99	28.88	27.38
60	8.95	9.51	8.65	9.04	22.33	21.28	23.65	22.42
72	9.08	9.65	8.77	9.17	24.32	23.18	25.75	24.42
84	14.67	15.59	14.17	14.81	20.24	19.29	21.43	20.32
96	15.28	16.24	14.76	15.43	19.85	18.91	21.01	19.92
108	15.80	16.79	15.27	15.95	21.63	20.61	22.90	21.71
120	16.35	17.37	15.79	16.51	19.82	18.88	20.98	19.98
132	21.45	22.79	20.72	21.65	19.80	18.87	20.97	19.88
144	27.50	29.22	26.56	27.76	20.13	19.18	21.31	20.21
156	33.57	35.67	32.43	33.89	18.61	17.74	19.71	18.69

**Table 6:** Determination of the amounts of proteins produced by *Pseudomonas taetrolens* using the Bradford method

Sample	Amount of proteins [μg/ml]
after I centrifuge	6,533
0.8 M (NH_4_)_2_SO_4_ after II centrifuge	34.5
1.2 M (NH_4_)_2_SO_4_ after III centrifuge	42
1.6 M (NH_4_)_2_SO_4_ after IV centrifuge	54.5
2.0 M (NH_4_)_2_SO_4_ after V centrifuge	58.6

**Table 7:** Changes of *Pseudomonas taetrolens* counts during the process of lactose oxidation to lactobionic acid

	Colony forming units [CFU/ml]
Inoculum after 24 h	2.8 × 10^9^
Bioreactor stationary method after 24 h	3.3 × 10^8^
Bioreactor continuous culture after 24 h	7.0 × 10^9^

The culture in the bioreactor may be run for a longer time to continue the analysis of changes in the contents of lactobionic acid and lactose, which results from the fact that the highest acid content was recorded at 144 h of culture. As it is a stationary culture, it may be expected that after some time, nutrients (lactose) may be depleted or microorganisms will be toxified by products of their own metabolism and will no longer produce lactobionic acid.

The next experiment consisted in the culture of microorganism, *P. taetrolens*, by continuous culture with constant inflow of fresh sterile whey to the bioreactor. Conditions found in the fermentation chamber were as follows: temperature 30 °C, pH 6.7, aeration rate 1 l/min and mixing 120 rpm. The experiment was repeated three times. At every 24 h, the volume of 500-ml culture was collected and it was supplemented with 500-ml sterilised whey. Samples were collected at every 12 h. Results are presented in the table below.

The above discussed investigations demonstrated that *P. taetrolens* DSM 21104 is capable of utilising lactose in metabolic processes as a carbon source. Growth of *P. taetrolens* demonstrated that under carbon-limited conditions, the cells were able to utilise lactose.

## Electrophoretic Analysis of Proteins in Cultures of *Pseudomonas Taetrolens*

Proteins searched for and analysed in cultures of *P. taetrolens* were exocrine enzymes

- lactose oxidase with molecular mass of 55 kDa [6]
- and lactonase with molecular mass of 28 kDa [10]

## Determination of the Number of Live Bacterial Cells of *Pseudomonas Taetrolens* in the Process of Oxidation of Lactose to Lactobionic Acid Using Koch’s Platelet Method

The number of live microbial cells of *P. taetrolens* was determined in the process of oxidation of lactose to lactobionic acid [9]. Inoculations were conducted from the inoculum culture at 24 h of incubation at 30 °C, from the stationary culture run in a bioreactor after 24 h of culture, and from the continuous culture run in a bioreactor at 24 h of culture.

## Discussion

Ahmad at al. 2004 have reported the toxicological investigations undertaken to evaluate the safety of a liquid enzyme concentrate, lactose oxidase, of this novel fungal oxidoreductase from *M. nivale*. The enzyme is expressed by a strain of *F. venenatum* and is produced by a submerged fermentation process and recovered by purification/concentration of the fermented culture broth. This introduction of the lactose oxidase gene into the recipient strain resulted in a *F. venenatum* production strain with significantly improved enzyme yields compared to what is obtainable by fermentation of the donor strain.

The enzyme is expressed by a strain of *Escherichia coli* and is produced by a submerged fermentation process and recovered by purification/concentration of the fermented culture broth. In our study, introduction of the glucose–fructose oxidareductase gene into the recipient strain resulted in an *E. coli* production strain with significantly improved enzyme yields compared to what is obtainable by fermentation of the donor strain.

Cheese whey was employed as a raw material for the production of lactobionic acid by *P. taetrolens* using a two-stage pH-shifted bioconversion strategy, and lactobionic acid production of 42.4 g/L with a 30 % volume seed culture inoculum was obtained after 32 h. Whey offers an alternative means to costly synthetic media with nutrient–mineral supplementation for lactobionic acid production. Further improvements concerning the influence of physical culture parameters on microbial behaviour as well as downstream processing are required for implementation of the process on an industrial level. [7]

The present study by Alonso et al. 2013 has demonstrated the feasibility of whey as an inexpensive source for lactobionic acid bio-production by *P. taetrolens* at an industrially relevant titer. A high-level titer of 180 g/L was obtained with a yield of 90 % via fed-batch cultivation carried out under co-feeding conditions. Moreover, the physiological responses of *P. taetrolens* cells were monitored through flow cytometry in order to assess the impact of different feeding strategies on bioprocess efficiency. High-yield bio-production of lactobionic acid was directly linked to the fully functional status of *P. taetrolens*, thus providing relevant information for successful industrial implementation.

## Conclusions

Lactobionic acid was produced as a result of enzymatic oxidation of lactose. Environmental factors affecting the yield of lactobionic acid were analysed, i.e. temperature and culture time. Optimal conditions for the production of this acid were found to be 48-h oxidation of lactose at 30 °C.

Certain irregularities were observed in the presented results, since in certain variants, the content of lactose increased. The content of oxidized sugar during culture should decrease due to the fact that lactose is transformed by exocrine enzymes in the first stage to lactobionic-δ-lactone, and subsequently to the molecule of lactobionic acid.

Conducted experiments confirmed that both the time and temperature influenced the production of lactobionic acid during bioconversion of lactose using the *P. taetrolens* bacteria.

The study also investigated the effect of inoculum concentration on the production of lactobionic acid as a result of oxidation of whey-derived lactose. The highest concentration of lactobionic acid during oxidation of whey-derived lactose at a temperature of 30 °C by microorganism *P. taetrolens* was obtained during 50-h oxidation of the medium, which contained 25 % addition of the inoculum, in which the count of live cells was 2.85 × 10^9^ CFU/ml.

Results of electrophoresis run by SDS-PAGE confirm in proteins salted out of the culture the presence of exocrine enzymes secreted by microorganisms *P. taetrolens*—i.e., lactonase with molecular weight of 28 kDa and lactose oxidase with molecular weight of 55 kDa.

Both the quantitative determination of proteins by Bradford’s method and electrophoretic determinations confirm the presence of enzymes produced by microorganisms *P. taetrolens*.
